# Infant Feeding

**Published:** 1883-11-20

**Authors:** 


### INFANT FEEDING.

**[Translated from the French, by Dr. E. Van Goidtsnoven, for the Southern Medical RECORD.—Le Journal Medical, Paris.]**

Professor Parrot, in a series of lectures on "Infant Feeding," dwells *a priori* on a few points very little known in the anatomy and physiology of the digestive tube of that age.

In the study of the buccal cavity he demonstrates the importance and utility which should be ascribed to the fatty gland of Bichat. This gland constitutes an oblique mass, slightly stretched at its extremities, situated at the junction of the anterior two-thirds with the posterior third of the buccal parietes. It is easily distinguished from the surrounding fatty tissue. Its size, after having increased up to the second or third year, gradually diminishes, and even changes in position. When under some pathological influence the child becomes emaciated, the gland loses its consistency and tends to resume its embryonic condition ; hence a notable perturbation in the act of suction. Professor Parrot believes that Bichat's gland plays an important role in this act, and that its existence and formation can be accounted for in the following manner :

As soon as the child begins to suck a vacuum is made within the buccal cavity which the internal walls of the cheek tend to fill. It is precisely above the alveolar arcades at the point where this tendency is effected, that Bichat's fatty gland can be found, the function of which evidently consists in facilitating the act of sucking by filling the vacuum which is formed at that moment. It may be admitted that in this instance the function is parent to the organ, and that repetition of functional activity necessitates this particular organic determination ; and that, should this function suspend its action for several thousands of years, this fatty gland would ultimately disappear, as now seems to be the tendency with the wisdom tooth, especially in the upper classes. At all events, it appears certain that when this organ has become atrophied under the influence of great emaciation, the act of sucking is rendered extremely difficult thereby.

The normal situation of the stomach in a child is very interesting to consider with respect to its functions. Whilst in man its axis is almost horizontal, it is nearly vertical in the infant, which is due to the organ not being yet fully developed. Hence, if the child be held vertically the food accumulates within the greater curvature; yet, it passes easily through the pylorus. If the child be held lying on its left side, this situation is particularly favorable for stomachal digestion, for the food is thereby retained longer in the viscus before reaching the pylorus. If, on the contrary, the child be held on his right side, the food passes almost instantly into the intestines.

Dorsal recumbence, with head slightly backwards, is very favorable to regurgitation, and this position more than any other is likely to produce asphyxia through the introduction of food in the trachea, so that with children who are subject to regurgitation, it should be deemed imperative to hold them for some time after being breast or bottle fed, in a vertical position, in order to allow the food to pass rapidly into the intestines.

With regard to the gastric juice secreted by the mucous membrane of the stomach, it has been ascertained that it contains pepsin already in the third or fourth month of intra-uterine life. In this instance it should be remembered that meconism is a product of digestion. But with the new-born the effects of gastric juice present a particularity very different from what takes place in the adult. As the infant continues to suck, even to its very last moments of existence, it follows that the gastric juice keeps on secreting; and as its peptonizing action is not exhausted even by death, it acts upon the parietes which are found softened much more frequently than in the adult. Thus when alimentary substances have found their way into the bronchial passages the lungs have been found entirely digested within a few hours following death.

[TO BE CONTINUED.]

